# Comparability of Results from Pair and Classical Model Formulations for Different Sexually Transmitted Infections

**DOI:** 10.1371/journal.pone.0039575

**Published:** 2012-06-27

**Authors:** Jimmy Boon Som Ong, Xiuju Fu, Gary Kee Khoon Lee, Mark I-Cheng Chen

**Affiliations:** 1 Department of Clinical Epidemiology, Tan Tock Seng Hospital, Singapore, Singapore; 2 Institute of High Performance Computing, Agency for Science, Technology and Research, Singapore, Singapore; 3 Department of Epidemiology and Public Health, National University of Singapore, Singapore, Singapore; 4 Duke-NUS Graduate Medical School, Singapore, Singapore; Public Health Agency of Barcelona, Spain

## Abstract

The “classical model” for sexually transmitted infections treats partnerships as instantaneous events summarized by partner change rates, while individual-based and pair models explicitly account for time within partnerships and gaps between partnerships. We compared predictions from the classical and pair models over a range of partnership and gap combinations. While the former predicted similar or marginally higher prevalence at the shortest partnership lengths, the latter predicted self-sustaining transmission for gonorrhoea (GC) and Chlamydia (CT) over much broader partnership and gap combinations. Predictions on the critical level of condom use (*C_c_*) required to prevent transmission also differed substantially when using the same parameters. When calibrated to give the same disease prevalence as the pair model by adjusting the infectious duration for GC and CT, and by adjusting transmission probabilities for HIV, the classical model then predicted much higher *C_c_* values for GC and CT, while *C_c_* predictions for HIV were fairly close. In conclusion, the two approaches give different predictions over potentially important combinations of partnership and gap lengths. Assuming that it is more correct to explicitly model partnerships and gaps, then pair or individual-based models may be needed for GC and CT since model calibration does not resolve the differences.

## Introduction

Mathematical models have been used to investigate the transmission dynamics of sexually transmitted infections (STIs) and assess the potential impact of public health interventions on both bacterial and viral STIs, such as the efficacy of screening measures on reducing Chlamydia prevalence [1], [2], and the effect of anti-retroviral therapy on incident HIV infections [3], [4].

Much of this modelling relies on variants of the “classical model” for STIs proposed by Hethcote and Yorke in the early 1980s [5]. The classical model describes populations of individuals with different rates of acquisition of new sexual partners (partner change rates, *R*). However, this modelling approach is not without its limitations. Firstly, the manner in which the model describes “sexual mixing” is possibly too simplistic. By comparing several modelling approaches, Eames and Keeling showed that accounting for contact network heterogeneities produces results that are different and likely more realistic [6]. A separate limitation relates to how the classical model treats all partnerships as instantaneous events, often with per partnership transmission probabilities that are independent of partner change rates. Some modelling studies have pointed out that individuals with higher partner change rates would likely have have have have shorter partnerships with fewer episodes of sexual intercourse within each partnership, and hence could theoretically have a lower probability of transmitting a given STI per partner than individuals with lower partner change rates and correspondingly longer partnerships [7], [8]. Much work with classical models now accounts for different partnership types (e.g. shorter casual partnerships versus longer stable partnerships), in effect assuming lower per partnership transmission probabilities for those with higher partner change rates [1], [9], [10], [11], but it is not clear if such modifications sufficiently counteract the limitations associated with modelling partnerships as instantaneous events.

Alternative approaches to treating partnerships as instantaneous events involves the use of pair models, which were proposed by Dietz and Hadeler in the late 1980 s [12], and individual-based models which followed in late 1990 s [13], [14]. Both approaches explicitly account for the duration of partnerships and the duration spent between partnerships (henceforth referred to as partnership (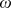) and gap (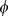) lengths respectively). Previous work by Chen et al. suggests substantial variability of partnership and gap lengths at the population level; by using a pair model, they also showed that stratifying a population into various categories of partnership and gap lengths has important implications on the transmission dynamics of gonorrhoea [15]. In addition, different contexts for STI transmission may be characterised by different partnership and gap length combinations. For instance, sex worker client interactions would be characterised by single episodes of sex between individuals, interspersed with extremely short gaps in the sex worker (of hours to days in the sex worker) and intermediate length gaps in the client (of several weeks to months) [16], [17]; romantic partnerships in young heterosexuals may comprise largely intermediate gap and partnership lengths on the order of a few months [18]; while the transmission context for heterosexual HIV in parts of Africa appears to involve both shorter as well as longer stable partnerships on the order of several months to years [19]. Accurately modelling the effect of partnership and gap lengths may hence be important for understanding the types of STIs likely to persist in different risk populations as well as predict the effect of potential interventions.

Explicitly modelling partnerships and gaps, as is done in pair and individual-based models, is intuitively more accurate, and work on pair models suggests that doing so results in different predictions from what would be expected from the classical model. Lloyd-Smith and colleagues suggested that this was particularly for bacterial STIs (which are mostly susceptible-infectious-susceptible (S-I-S) type infections with a shorter duration of infectiousness) than for viral STIs (which are mostly susceptible-infectious (S-I) type infections with a longer duration of infectiousness) [20]. However, Kretzschmar and Dietz also pointed out that, for the same set of model parameter values, different epidemic growth rates and steady-state prevalence (*π^s^*) can result when modelling S-I type pathogens with the two different model formulations [21]. However, neither work considered whether the results would be more similar if model outputs had been calibrated to observed data by allowing model parameter values to vary, as is done in much modelling work (e.g. [1], [4], [9], [22]). One recent paper suggests that, when modelling Human Papilloma Virus, both the pair and classical model formulations produce reasonably similar predictions on the impact of vaccination, provided transmission rates are first calibrated to match the same empirical data on pre-vaccination prevalence [23]. However, it remains unclear if model calibration can reduce the discrepancy in predictions when applied to other STIs, and for what types of sexual behaviour (framed in terms of partnership and gap lengths). In particular, re-infection within partnerships can prolong the infectious duration of S-I-S type infections, a phenomena not adequately accounted for within the classical model [24].

In this work, we aim firstly to identify the context, both in terms of disease types and sufficiently broad combinations of partnership and gap lengths, when the classical model̀s assumption of instantaneous partnerships may produce different predictions from those derived when explicitly modelling partnerships and gaps. We do so by using simplified versions of the classical and pair models to predict *π*, the prevalence of infection, and *C_c_*, the critical amount of condom use needed to prevent self-sustaining transmission. This was done for model parameters depicting gonococcus (GC) and Chlamydia trachomatis (CT) infections, as examples of S-I-S type infections with different reproductive potentials [25]. We also modelled HIV in the absence and presence of co-factor enhancement, represented respectively as an S-I type infection with lower and higher estimates of per-sex-act transmission probability [26]. Secondly, by assuming that explicitly modelling partnerships and gaps is more correct, we investigate whether and when model calibration for the classical model reduces the divergence in predictions for each partnership and gap length combination. To approximate situations when model outputs are calibrated to disease prevalence data, we first altered one key infection parameter by an adjustment factor (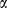) so that the classical model could reproduce the prevalence predicted by the pair model, then estimated again the predicted critical condom use (*C_c_*') using the classical model with the “calibrated” parameter. We conclude by pointing out situations where predictions from the classical and pair model diverge substantially in spite of model calibration, as these contexts would be where more complex modelling approaches such as pair and individual-based models may be needed.

## Results

**Figure 1:**
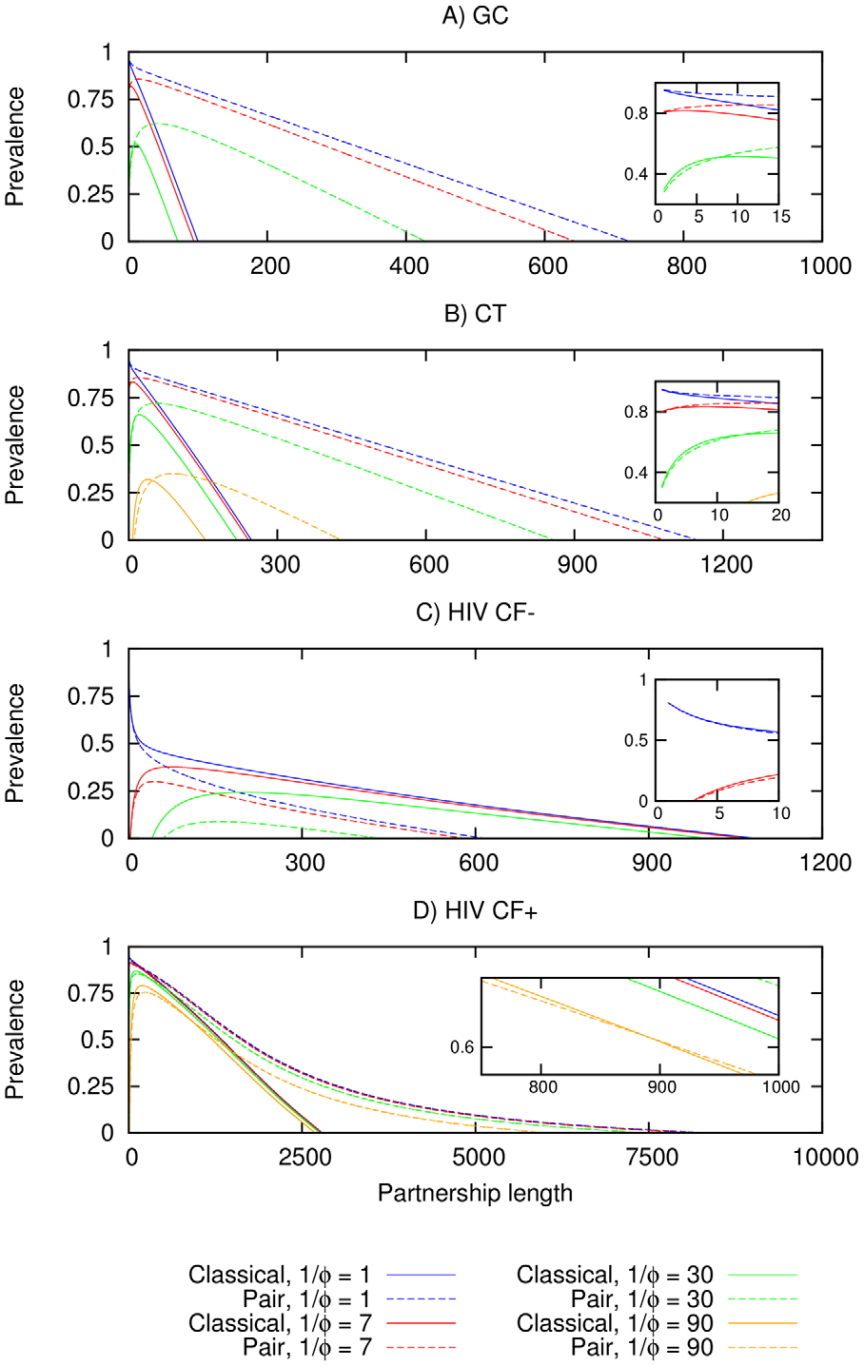
Predictions from the classical and pair model formulations for the steady-state *π^s^*of GC/CT (A and B), and the peak *π^p^* of HIV with and without cofactor enhancement (C and D). The horizontal axes give partnership length in days while the vertical axes give *π*. The different lines denote predictions from using gap lengths (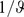) of 1 day, 7 days, 30 days and 90 days. The inset in each figure magnifies crossover point, if any, in the region where the classical and pair models diverge in *π* predictions. Models in (A) and (C) are unable to provide predictions at a gap length of 90 days.

Using the baseline parameters in Table 1, Figure 1 contrasts the predictions from the pair and classical model formulations for the steady-state prevalence (*π^s^*) for GC and CT, and the peak prevalence (*π^p^*) for HIV with and without cofactor enhancement over various combinations of partnership and gap lengths (for an explanation of why peak prevalence is used in HIV, see methods and Figure S1). For GC and CT, we see that predictions on *π^s^* begin to diverge substantially for anything but very short partnership lengths (less than 10 days). For the longer gap lengths (30 days for both, and 90 days for CT only), the classical model gives a higher value of *π^s^* than the pair model at lower partnership lengths and vice-versa as partnership lengths increase, the cross-over occurring at 8 days for a gap length of 30 days in GC, and at 14 and 48 days for gap lengths of 30 and 90 days respectively for CT. The lower values of *π^s^* at combinations of shorter partnership lengths and longer gap lengths in the pair model is partly due to the explicit inclusion of the pair-formation process which reduces opportunities for infectious contacts (for an elaboration, see discussion and [27]); at longer partnership lengths, this effect is offset by the potential re-infection within intermediate to longer partnerships, which is accounted for in the pair model but ignored in the classical model. For S-I type infections, where re-infection does not apply, the inclusion of the pair-formation process results in the pair model predicting lower values of *π^p^* for HIV without cofactor enhancement (HIV CF-) throughout. For HIV with cofactor enhancement (HIV CF+), however, a crossover in the predictions of the pair and classical models occurs (at 299 and 899 days for gap lengths of 30 and 90 days respectively). In this case, HIV-induced mortality is resulting in additional pair separation of HIV concordant pairs in what would otherwise be very long and stable partnerships, an effect which was ignored classical model.

**Table 1:** Model parameters.

Parameter [Reference]	Symbol	Value
Frequency of sex [9], [15]	*ζ*	1 in 3 days
Sexually active life-span	μ	35 years
Efficacy of condom use [1]	ε	0.9
Duration of infectiousness, gonorrhoea [32]		
- symptomatic males that receive treatment	1*/σ*_1_ *^M^*	13 days
- symptomatic females that receive treatment	1*/σ*_1_ *^W^*	20 days
- males and females who do not receive treatment	1*/σ*_2_ *^M^*, *1/σ*_2_ *^W^*	185 days
Proportion which are symptomatic and receive treatment, gonorrhoea [38]		
- male	*θ*_1_ *^M^*	0.59
- female	*θ*_1_ *^W^*	0.36
Per sex act transmission probability, gonorrhoea [42], [43]		
- male-to-female	*β^M^*	0.50
- female-to-male	*β^W^*	0.25
Per sex act transmission probability, Chlamydia ([45] see text)		
- male-to-female	*β^M^*	0.33
- female-to-male	*β^W^*	0.06
Duration of infectiousness, Chlamydia [44]		
- symptomatic males and females that receive treatment	*1/σ*_1_ *^M^*, *1/σ*_1_ *^W^*	35 days
- males and females who do not receive treatment	*1/σ*_2_ *^M^*, *1/σ*_2_ *^M^*	300 days
Proportion which are symptomatic and receive treatment, Chlamydia [38]		
- male	*θ*_1_ *^M^*	0.09
- female	*θ*_1_ *^W^*	0.24
Per sex act transmission probability, HIV without cofactors [9]		
- acute stage	*β*_1_	0.0107
- chronic stage	*β*_2_	0.0008
- advanced stage	*β*_3_	0.0042
Per sex act transmission probability, HIV with cofactors ([26] see text)		
- acute stage	*β*_1_	0.0428
- chronic stage	*β*_2_	0.0032
- advanced stage	*β*_3_	0.0168
Duration of each of HIV stages [9]		
- acute stage	1/*σ*_1_	2.5 months
- chronic stage	1/*σ*_2_	7.59 years
- advanced stage	1/*σ*_3_	2.0 years

**Figure 2:**
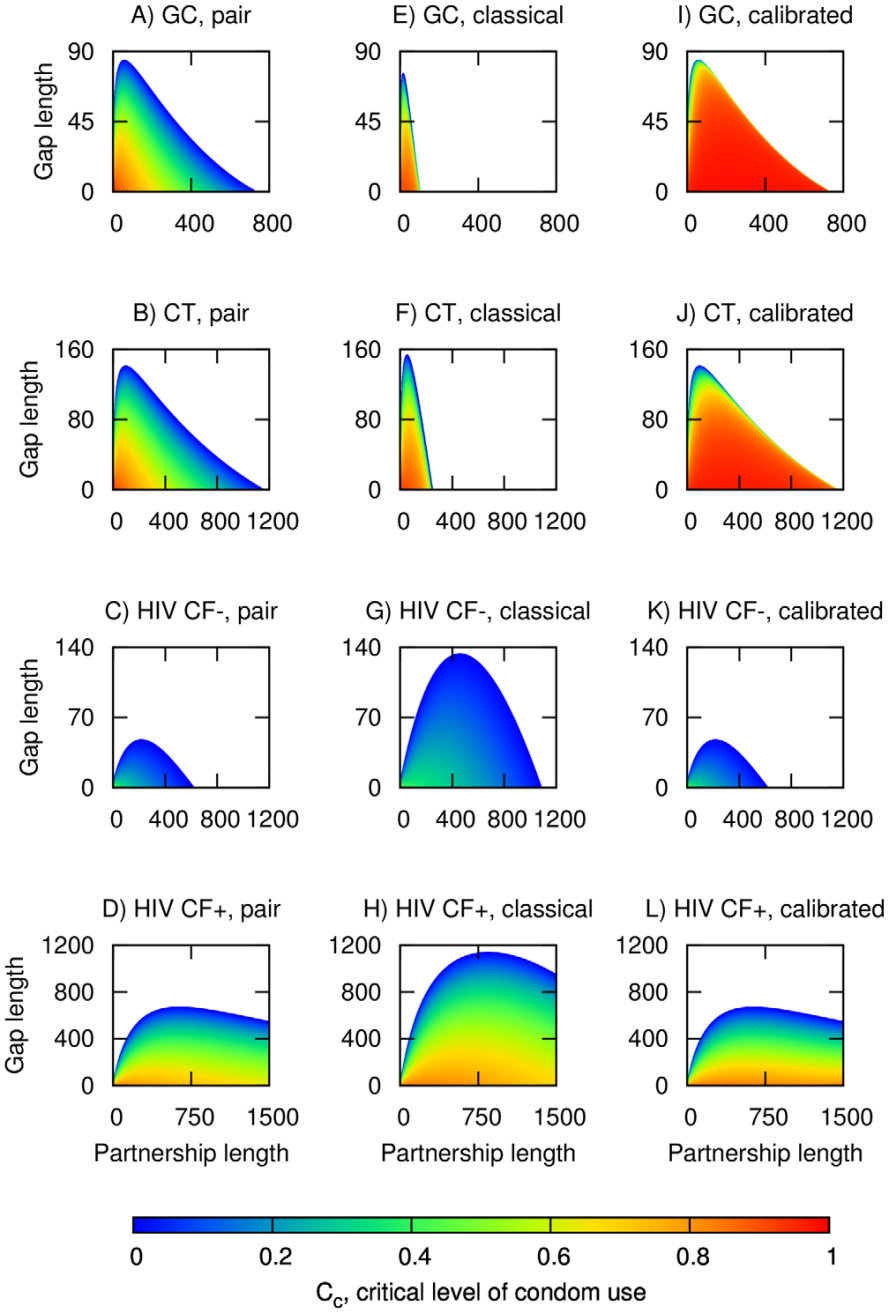
Critical level of condom use (*C_c_*) predicted to prevent self-sustaining GC/CT and HIV transmission for the pair (A to D), classical uncalibrated (E to H), and classical model following calibration of *π* to the pair model output (I to L). The horizontal axes give partnership length in days while the vertical axes give gap length in days. *C_c_* values are denoted by a gradient of colours as indicated; values of 0% demarcate the most extreme combination of partnership and gap lengths which supports self-sustaining transmission, while values above 100% (up to a theoretical maximum of 111% since condoms are assumed to be only 90% effective in preventing transmission) show partnership and gap combinations where consistent condom use is insufficient to prevent self-sustaining transmission.

Figure 2 explores *C_c_*, the critical level of condom use predicted to prevent self-sustaining transmission (i.e. so that effective reproduction number is less than 1) when using the pair model (Figures 2A to D), the classical model (Figures 2E to H), and a classical model which has been calibrated to give the same values of *π^s^* and *π^p^* as the pair model (2I to L); for simplicity, condoms were assumed to have 100% efficacy so that 100% condom use would prevent all transmission. Predicted *C_c_* values are given by a colour gradient from 0% (blue) to 100% (red); *C_c_* values of 0% also demarcate the most extreme combination of partnership and gap lengths which can support self-sustaining transmission. For all the four infection parameter sets, both the pair and classical models predict that the longest permissible gap length occurs at some partnership length between the extremes of values modelled. However, the classical model predicts a much more restricted range of partnership and gap combinations for self-sustaining transmission of GC and CT than the pair model. Both approaches predict maximum permissible gap lengths of similar magnitude, with the values for CT being substantially longer than for GC. However, even with extremely short gap lengths, permissible partnership lengths extend only up to 100 and 248 days respectively in the classical model. On the other hand, the pair model predicts that intermediate to longer partnership lengths of up to 724 day for GC and 1150 days for CT could still support self-sustaining transmission. For HIV without cofactor enhancement, the reverse is true. The classical model gives a wider range of permissible partnership lengths of up to 1000 and gap lengths of up to 140 days, while the corresponding values for the pair model are 617 and 48 days. For HIV with cofactor enhancement, the maximum permissible gap length is less in the pair model as compared to the classical model (671 vs. 1138 days), but the maximum permissible partnership length is much greater (8390 days vs. 2786 days; extends into the truncated area in figure 2D and 2H) due to the additional pair separation from HIV-induced mortality that arises when HIV involves long stable partnerships.

With regards to the critical level of condom use which prevents self-sustaining transmission, both the pair and classical model formulations predict the same general pattern of decreasing *C_c_* with increasing gap length, but the exact predictions differ. In the overlapping combinations where both models predict self-sustaining transmission, the classical model generally predicts higher *C_c_* values; for example, for GC at a partnership length of 30 days and a gap length 30 days, the corresponding predictions for *C_c_* are 0.7704 and 0.6984 in the classical and pair models respectively.

**Figure 3:**
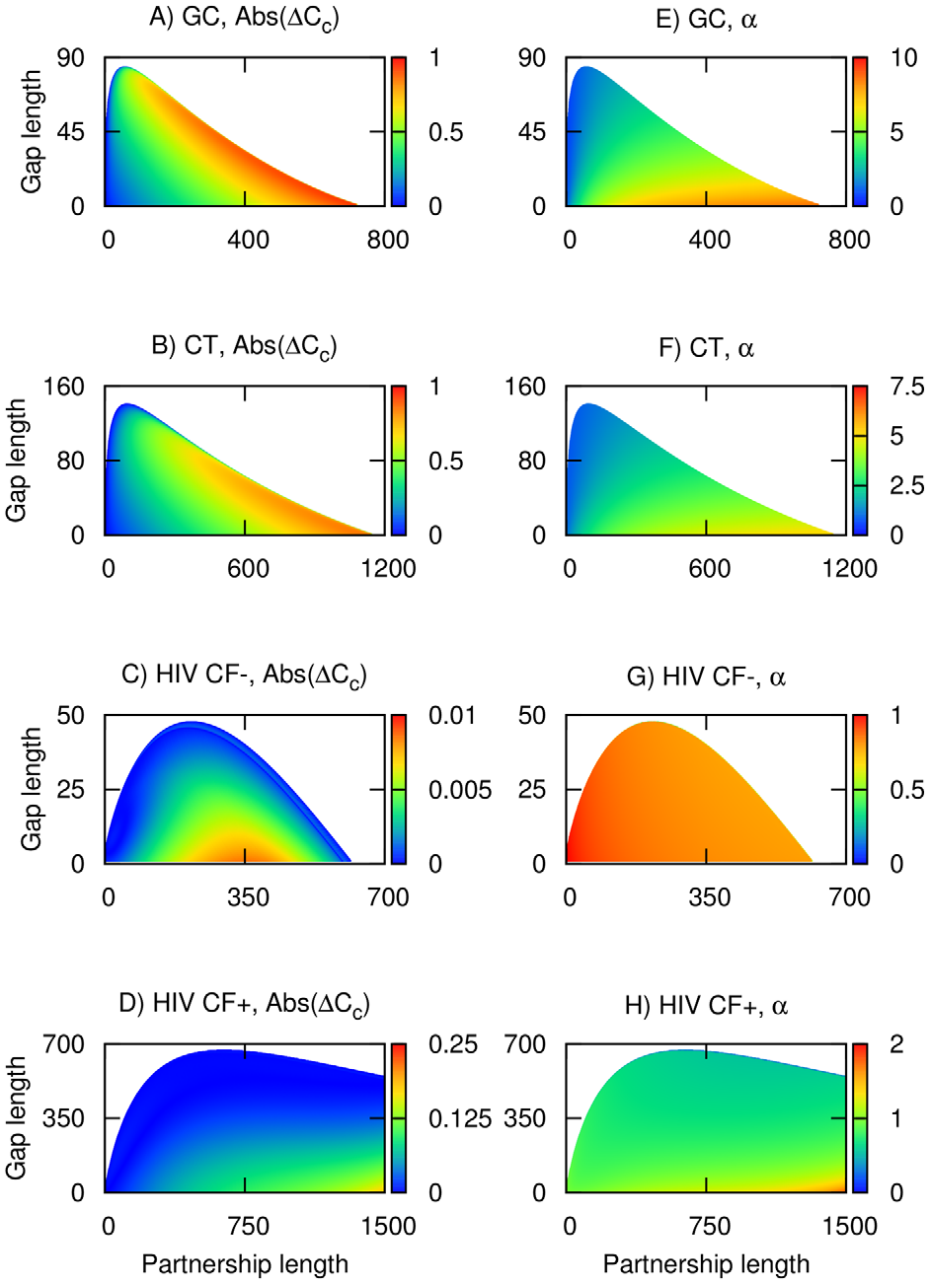
Absolute difference in predicted critical level of condom use (Abs(Δ*C_c_*)) for GC/CT and HIV with and without cofactor enhancement (A to D), with its corresponding adjustment factor, 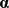
(E to H). The horizontal axes give partnership length in days while the vertical axes give gap length in days. Abs(Δ*C_c_*) is computed from the absolute difference in the corresponding values from Figure 2. Coloured bars in the left (A to D) and right (E to H) panels give the values of Abs(Δ*C_c_*) and 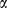 by the respective gradient of colours.

Assuming the pair model more accurately predicts disease prevalence and the partnership and gap combinations where self-sustaining transmission is possible, the classical models for GC and CT were calibrated to give the same values of *π^s^* as the pair model by adjusting the duration of the non-care-seeking infections (Figures 2I to 2J). After calibration, the classical model now predicts higher *C_c_* values at longer partnership lengths, the effect being more pronounced for GC than for CT. The calibrated classical model also predicts that close to 100% condom use would be required to prevent self-sustaining transmission for a wide range of partnership and gap combinations. The divergence between the predictions of the pair and calibrated classical model is elaborated on in Figures 3A and B, which highlight the areas where the absolute difference in predicted *C_c_* values is close to 100% for GC (i.e. combinations when *C_c_* approaches the maximum of 100% in the calibrated model and is close to 0% in the pair model). However, Figures 3E and F show that the corresponding adjustment factors used in the calibrated classical model are at the edge of plausibility, since 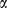 approaches 10 for GC, and 7.5 for CT at longer partnership lengths, in effect assuming infectious periods in the realm of several years. In contrast, after the classical model for HIV was calibrated by adjusting per sex act transmission probabilities, predicted *C_c_* values are fairly close to those from the pair model (Figures 2K and 2L); differences are less than 1% for HIV without cofactor enhancement and less than 10% for most partnership and gap combinations for HIV with cofactor enhancement (Figures 3C and 3D). Moreover, adjustment factors are fairly close to 1 for most combinations of partnership and gap lengths (Figures 3G and 3H). However, for HIV with cofactor enhancement, the calibrated model could only be extended up to partnership lengths of about 1500 days, as the assumption of higher adjustment factors would have resulted in values for per sex act transmission probabilities exceeding 1 (for the primary stage of HIV infection), and it was hence not possible to replicate the dynamics from the pair model for long stable partnerships.

## Discussion

It has been hypothesized that STIs can broadly be divided into two groups based on their transmission dynamics: those with short infectious periods but high transmission probabilities (mostly bacterial STIs, with S-I-S dynamics) and those with long infectious periods but low transmission probabilities (mostly viral STIs, with S-I dynamics) 28]. Using parameters for gonorrhoea and Chlamydia to represent the former, and parameters for HIV (with and without cofactor enhancement) to represent the latter, we compared the traditional classical model against results from the pair model and found that the two model formulations lead to very different results on predictions about disease prevalence, partnership and gap length combinations that support transmission, and the levels of condom use needed to prevent self-sustaining transmission. Calibrating the classical model to give similar outputs for *π^s^* and *π^p^* as the pair model fails to reconcile the predictions for an S-I-S type infection with gonorrhoea and Chlamydia-like parameters, but does reduce the differences in predictions on condom use for an S-I type infection like HIV.

For bacterial or S-I-S type infections, Lloyd-Smith and colleagues had previously demonstrated that the two model formulations diverge greatly in predicting epidemic growth rate under a simplified situation which varied the partner change rate while assuming the partnership and gap lengths to be of equal duration [20]. In our case, we independently varied partnership and gap lengths while looking at predictions on steady-state prevalence (*π^s^*) and condom use, hence providing several additional insights with key implications. Firstly, it is worth re-emphasizing that the pair model identifies a broader spectrum of behaviours as populations potentially capable of sustaining the transmission of S-I-S type infections; this would include individuals with intermediate partnership lengths (on the order of a few months) combined with short gaps in gonorrhoea (less than 3 months), and short to intermediate gap lengths (up to about 5 months) in Chlamydia [15], [24]; the potential transmission of Chlamydia in populations with longer gap lengths may explain why CT has a wider distribution range in the population than GC [29], [30], [31]. Secondly, we show that, across the range of partnership and gap lengths investigated, discordance in predictions occurs mainly from intermediate to longer partnership lengths, with the discordance being greater for gonorrhoea which has a shorter infectious period than Chlamydia. This helps identify the combination of disease and behavioural contexts where both modelling approaches would yield similar results, and where they might diverge. Thirdly, our work shows that fitting S-I-S models to data will not resolve the discrepant quantitative predictions which arise from the choice of model formulation. Logically, either one or both the model formulations are failing to adequately represent reality. In particular, Garnett and colleagues have previously pointed out that the classical model applied to gonorrhoea can give predictions that are unrealistically sensitive to small changes in parameter values, and our work adds weight to the previous call for caution on the interpretation of such results [32]. Assuming that the areas of divergence between the two approaches does highlight the limits of the classical model in a given disease context, then given the restricted range of partnership lengths (less than a few weeks) for gonorrhoea, it is difficult to think of significant applications in the heterosexual context for the classical model other than client sex worker interactions. On the other hand, since results for Chlamydia are still reasonably similar for partnership lengths up to a couple of months, the classical framework may still be adequate for modelling Chlamydia transmission in partnerships amongst at-risk heterosexual youths like those described by Bearman et al. [18].

With regards to viral and other STIs with S-I dynamics, our work shows that, for a less infectious pathogen like HIV without cofactor enhancement, the classical model predicts a higher peak prevalence (*π^p^*) and self-sustaining transmission over a wider combination of partnership and gap lengths. This can be explained by the reduced opportunities for transmission imposed by the pair formation mechanism; as previously pointed out by Kretzschmar [27], susceptible individuals who are single or in stable partnerships with other susceptible individuals, as well as infectious individuals paired with other infectious individuals are all excluded from the transmission process (which occurs only in pairs where one individual is infectious and another is susceptible). This leads to slower epidemic growth rates in the pair model [21] and hence a lower value of *π^p^* for HIV, as well as the need to assume higher pair formation rates to allow self-sustaining transmission. However, we also show that, while we get similar results for HIV with cofactor enhancement at shorter partnership lengths, the pair model predicts higher values of *π^p^* and greater potential for self-sustaining transmission at longer partnership lengths as compared to the classical model; this results from the dominance of HIV-induced mortality on pair separation at longer partnership lengths which is unaccounted for in the classical model. While it is comforting to know that model calibration seems to resolve the discrepant predictions between the pair and classical models, it must be remembered we are adjusting the classical model using individually derived adjustment factors for each partnership and gap combination, while in practice most model fitting would involve using some average adjustment factor for disease transmissibility or other parameter value [23]. The classical model may thus underestimate the role of long-term stable partnerships in propagating HIV even when calibrated to data. Other additional differences have been highlighted by others, including the prediction of higher epidemic growth rates and higher estimates on the contribution of acute infectious stages by the classical model as compared to the pair model [21], [33].

As with any modelling work, several limitations and assumptions must be acknowledged. Firstly, we must reiterate that our analysis only highlights the difference in predictions between the pair and classical models, and does not prove which model formulation is more accurate. It has been argued that the pair model is a more correct representation of STI dynamics [12], [20], but what is modelled here is a serially monogamous population rotating through a fixed partnership and gap length combination in isolation and in perpetuity. In reality, populations comprise individuals with a heterogeneous mix of behaviour that changes with successive partnerships. In addition, concurrent partnerships would relax the constraint on individuals to have contacts with only one partner at a time, and a pair model accounting for concurrency may be closer in dynamics to the classical formulation for S-I type infections. On the other hand, concurrent partnerships increase the potential for re-infection within partnerships, and may thus exaggerate the differences between the two model formulations for S-I-S type infections. One question is how significant re-infections are in prolonging the duration of S-I-S type infections. Several studies suggest that some re-infections arise from the same source partner [34], [35], and the effectiveness of expedited partner therapy in reducing repeat infections emphasizes their importance to such STIs [36], [37]. However, stochastic extinction, partial immunity and treatment of partners would in reality reduce the effect from re-infections within partnerships. Concerns may also be raised on the parameters describing both the sexual behaviour of the population and the natural history of the various STIs modelled. For instance, in the absence of sufficiently detailed data on how the frequency sex might vary with partnership duration, we assumed that sex occurred at the same frequency in all partnership lengths, although this is unlikely to be the case in reality; results from the classical and pair models would be less divergent if sex is less frequent than we had assumed in longer partnerships, and vice versa if the reverse is true. Also, for infection parameters, there have also been no direct estimates for the per sex act transmission probability of Chlamydia that we know of, and it has been difficult to accurately measure the duration of infectiousness, as well as the proportion of incident infections that are asymptomatic or do not receive treatment for both gonorrhoea and Chlamydia; this is particularly since such infections would not be accurately represented in clinic-based data. Our study used estimates for the proportion of incident infections which are symptomatic and receive care as derived by Farley et al. [38], which was based on a case-finding type strategy, with its inherent limitations; in particular, the Chlamydia infections in men receiving treatment with symptoms is lower than what has typically been assumed in other studies. Also, our approach of calibrating the classical model to the pair model through an “adjustment factor” could be criticised; this resulted in the need to assume implausible parameter values for the S-I-S infections, beyond the bounds of what would normally be used in modelling studies. However, we did this not because we intended to use such re-scaled parameters to model the infection in the classical framework, but more to illustrate the dangers in calibrating an inappropriate model, and to identify situations where model calibration might fail to help based on the assumption that the pair model was more correct. An alternative approach used by others would be to present the threshold values for transmission probabilities or infectious duration for the competing modelling approaches [39], and highlight the partnership and gap combinations where the divergence between the two models occurs; this would have avoided passing judgment as to which approach is more correct. Finally, while a deterministic pair model was sufficient as a means of identifying situations where the classical model is most likely inadequate, descriptions of sexual networks ranging from those involving heterosexual youths [18] to sex worker client encounters [40] have revealed high levels of complexity and heterogeneity, and other work has shown that such network heterogeneities have an important effect on transmission dynamics that is inadequately approximated by both the pair and classical models [6], [41]. These observations, along with the importance of modelling partnership and gap durations demonstrated in this paper, adds to the impetus for developing efficient individual-based models which can simultaneously account for both factors.

In summary, our work suggests that outputs from classical and pair model formulations are in conflict for a range of gap and partnership combinations which could possibly support the transmission of some common STIs. Model calibration may resolve the discrepancies for S-I type infections, but only for the very shortest partnerships in S-I-S type infections. If we accept that the actual transmission process is better modelled by the pair rather than the classical model, then our findings emphasize the need to move beyond measuring partner change rates to account for partnership and gap behaviours, and to adopt STI modelling approaches which accounts for the effect of partnership and gap lengths on STI transmission.

## Methods

### Overview of model structure, infection parameters and notation used

Both the classical and pair models were deterministic compartmental models depicting a heterosexual population, 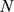, with an equal number of males and females, with additional compartments to represent different infection states; the pair model also included compartments to represent pairs with different infection state combinations. The population was assumed to have a finite sexually active lifespan of duration (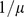), so that turnover of this sexually active population occurs; the sexually active lifespan was assumed to be 35 years. Individuals leaving the sexually active pool are replaced at the same rate by uninfected individuals. In all formulas, superscripts denote gender (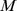
for males and 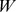 for females), while subscripts are used to denote the infection state.

Gonorrhoea and Chlamydia (GC/CT) were both depicted as susceptible-infectious-susceptible (S-I-S) type pathogens, where the infection states were 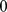 for susceptible, 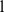
for symptomatic infections that receive treatment, and 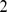
for infections which either result in symptoms but do not receive treatment, or are completely asymptomatic; the proportion (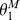, 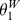) who are symptomatic and receive treatment have shorter infectious periods, (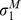, 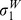), while the remainder (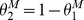, 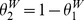) have longer infectious periods (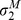, 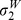). Individuals recover without mortality and are immediately susceptible to re-infection.

HIV was depicted as a susceptible-infectious (S-I) type pathogen with three successive stages of duration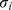, where 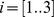 represent primary, chronic, and advanced HIV infection respectively. Susceptible individuals (denoted by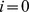) enter the primary infection stage and progress through stages chronic, and advanced HIV; individuals with advanced HIV are removed by HIV-induced mortality, and are not replaced.

The corresponding parameter values used are in Table 1. Sex within partnerships was assumed to occur at a frequency of once in 3 days, while condoms were assumed for simplicity to be 100% efficacious in preventing transmission; these are values similar to those assumed in other studies [1], [9], [15]. For gonorrhoea, we used the per-sex-act transmission probabilities estimated from historical studies [42], [43] and the same infectious durations proposed by Garnett et al. [32]. The proportions which are symptomatic and receive treatment for gonorrhoea follows the estimates from a study by Farley and colleagues; we also used the corresponding estimates for Chlamydia from that study [38]. While there are some estimates on the infectious durations of Chlamydia [44], there are no direct estimates of per-sex-act transmission probabilities for Chlamydia; we used the data from Lycke et al. [45] to obtain some estimate for this parameter, with details being described in the section on Transmission probabilities for Chlamydia.

Parameters used to depict the durations of acute, chronic and advanced stages of HIV infection, and the corresponding per-sex-act transmission probabilities in these stages follow those proposed by Abu-Raddad et al. [9], [46]. We considered a scenario for HIV with per-sex-act transmission probabilities which were four times higher, as cofactors such as ulcerative genital disease have been estimated to enhance transmission by such an amount [26].

### Pair model

The pair model depicts a serially monogamous individuals cycling through the unpaired and paired states. The letter 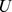 with the corresponding superscripts and subscripts was used to depict the number of unpaired individuals of a particular gender and infection state, e.g. for gonorrhoea, 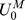
would be the number of susceptible unpaired males. The letter 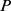 with two subscripts separated by a comma give the corresponding infection state for the male followed by the female member of pair, e.g. for HIV, 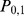
is a susceptible male paired with a female with primary HIV infection.

Pair formation occurs when opposite gender individuals transit from the unpaired to the paired state at a rate (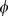) inverse to the specified gap length, and pair separation occurs when paired individuals return to the unpaired state at a rate (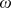) inversely related to partnership lengths; additional pair separation occurs from turnover of sexually active individuals and HIV-related mortality, where one member is removed and the surviving member is returned to the unpaired state. Transmission potentially occurs at the instant of pair formation between an infected and an uninfected individual, in accordance with the infection state specific per-sex-act probability of transmission (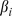) modified by the proportion of sex acts protected by condom use (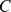) and condom efficacy (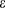). For instance, for HIV:
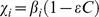



Within pairs between a susceptible and infectious member, potential transmission continues at a rate 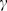; this is based on the chance of avoiding infection after the number of sex acts, 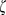, that occurs per unit time, and the respective transmission probabilities modified by condom use (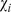), so that:
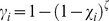



Disease transmission results in transitions between the pairs with different infection state combinations, as do progression of HIV infection and recovery from GC/CT in the respective pair models; progression of and recovery from infection also applies to individuals in the unpaired state. Pairs of a particular infection state combination form at a rate proportionate to the availability of unpaired opposite sex individuals from the respective infection states, while pair separation returns individuals to the respective compartments by infection state and gender.

In the GC/CT model, 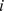
and 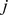 denote the three possible infection states (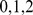) of the male and female member of the pair respectively, 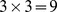
compartments for pairs and 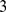 compartments for unpaired individuals of each gender as follows:
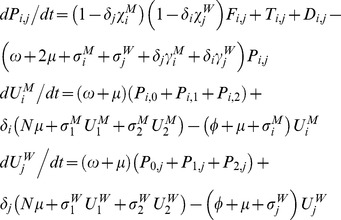




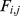
, 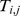 and 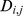 are transitions resulting from pair formation, transmission of infection and disease recovery, where:
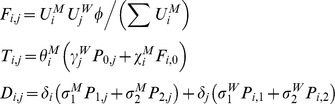



In all the above, 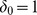
and 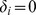 for all other values of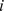, so that certain expressions are active only for the relevant pair compartments; also, 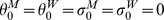 and 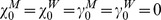 since individuals in the susceptible state do not recover from or transmit the infection. In addition, 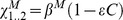, 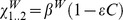, 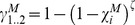, and 
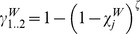
.

Disease prevalence, *π,* was expressed as
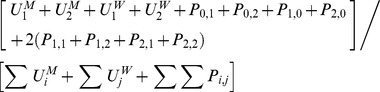



In all analyses, steady-state GC and CT prevalence (*π^s^*) was used.

In the HIV, there are 4 possible infection states (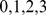) with a total of 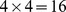 combinations of pairs and 4 compartments for unpaired individuals of each gender, as follows:
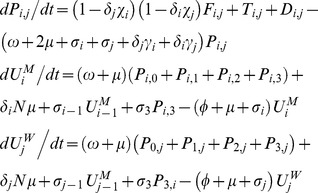




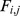
 is defined similarly as for GC/CT, while 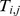 and 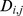 are defined differently since all new infections enter via stage 1 and then experience disease progression, as follows:
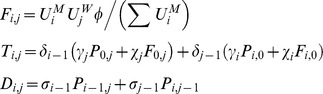



In all the above, 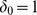
and 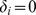 for all other values of 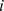; we also defined several dummy variables and parameters that are set to 0, including 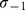, 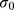, 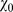, 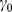, 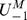, 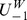, 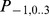 and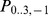.

Disease prevalence, *π,* was expressed as
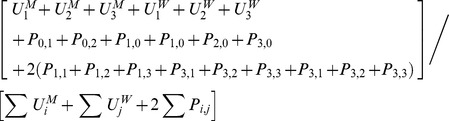



In the case of HIV, modelling predictions on steady-state prevalence will vary depending on assumptions about replacement of at-risk individuals removed from the system due to HIV-related mortality; moreover, most epidemics are still evolving, and have not reached their steady-state prevalence, although the prevalence in some geographical areas and population groups may have passed their peak. We therefore used the peak value of prevalence (*π^p^*) predicted by the model in all our analyses.

### Comparable formulation of the classical model

The classical model treats partnerships as instantaneous events with a per-partnership transmission probability (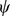) commonly computed by estimating the chance of avoiding infection for the total sex acts in partnerships of a given length, regardless of the duration of the infectious stage (e.g. [9], [10]). To apply this, we assumed that sex occurs once upon partnership formation then at frequency 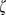 till the partnership ends. For instance, for each infectious stage in the HIV model:
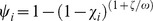



where 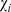 is the stage specific per-sex act transmission probabilities as modified by condom use; in the classical model formulation of HIV:
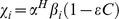



where 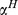 is an “adjustment factor” which has a default value of 1 but can be altered to change the value of *π^p^* from the classical model (as explained in a subsequent section on calibrating classical model to pair model outputs).

The partner change rate (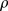) for the classical model is approximated by the inverse of the “cycle length”, which is the time taken to cycle through successive gaps and partnerships, i.e.:
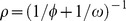



If 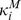 and 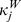 are the number of males and females of the respective infection state, then for gonorrhoea and Chlamydia:
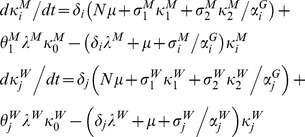



where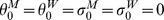, and as with the pair models, 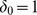
and 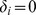 for all other values of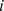; 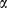 is again an “adjustment factor”, and in this case we fix 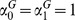 while adjusting 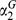 from the default value of 1 to adjust the output of *π^s^* from the classical model, since only the duration of non-care-seeking infections is altered (more detailed explanation to follow later).

The symbols 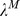 and 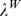 define force of infection acting on males and females respectively, where:
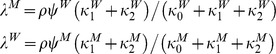



where 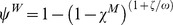, 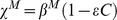 and likewise for 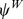 and 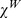.

Disease prevalence, *π,* was expressed as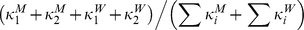, with the steady-state GC/CT prevalence, *π* ^s^, used in all analyses.

For HIV:
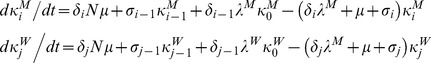
where several dummy variables and parameters that are set to 0, including 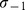, 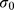, 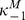 and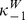. Again, 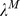
and 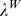 define force of infection acting on males and females respectively, where:



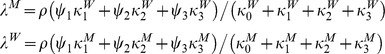
where 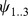 are as defined previously and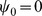.

Disease prevalence, *π,* was expressed as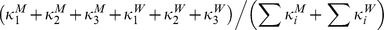. As with the HIV pair model, all analyses refer to the peak prevalence, *π^p^*, predicted by the model.

### Estimating the critical level of condom use

We also determined for the pair model and the classical models the “critical level of condom use” (*C_c_*). This parameter represents the proportion of sex acts which would need to be protected (e.g. by condom use, or some other similar intervention) to prevent the infection from spreading.

### Calibrating classical model to pair model outputs

It is not uncommon in STI modelling work (e.g. [1], [4], [9], [22]) to calibrate model outputs to observed data by allowing model parameter values to vary. Our aim was to see if transmission dynamics in sub-populations, as characterised by particular combinations of partnership and gap lengths, could be adequately modelled with the classical approach. Since we lack real data on sub-population specific for the different diseases, we instead start with the assumption that the pair model more accurately depicts transmission dynamics. We then altered one key infection parameter in the classical model by an adjustment factor (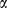) so that it could reproduce the prevalence predicted by the pair model. Then, using the classical model with the “calibrated” parameter, we re-estimated the predicted critical condom use (*C_c_*') for that partnership and gap length combination.

In the case of GC and CT, model fitting often uses estimates of prevalence (e.g. [1], [47]), so model fitting was performed to minimize the difference in the value of *π^s^* (see Figure S1A). Re-infection extends the infectious period of an S-I-S pathogen in the pair model [24], and there is considerable uncertainty in estimates on the duration of non-care-seeking infections; we therefore adjusted the value of *π^s^* for GC/CT by altering this parameter through an adjustment factor (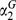).

In the case of HIV, there has been a wide variation in estimates on the per-sex-act transmission probabilities [48]. We therefore calibrated the classical model to give the same value of *π^p^* (see Figure S1B) as obtained from the pair model by simultaneously multiplying the transmission probability across all 3 infectious stages using the same adjustment factor (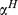).

### Model implementation and solutions

At the steady-state prevalence for GC and CT, the value in each of the compartments does not change, i.e.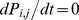, 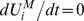 and 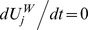 in the pair model, and 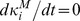 and 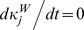 in the classical model. We solved the above sets of 15 and 6 simultaneous equations in the pair and classical models numerically to find the respective steady-state prevalence, *π^s^*, for each parameter set, as well as to obtain the adjustment factor (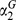) for a calibrated pair model which would give the same value of *π^s^* as the pair model; we also verified using the dynamic version of the model that the simulated values of *π* approached the calculated values of *π^s^* after 100,000 model days. For HIV, the peak prevalence, *π^p^*, was obtained by simulation, as was the adjustment factor (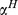). The solution for the critical level of condom use (*C_c_*) was also found numerically. All models implemented using the Java programming language version 1.6.0_26.

### Transmission probabilities for Chlamydia

We focused on the case-contact pairs where the index was co-infected with Chlamydia and gonorrhoea described by Lycke et al. [45]. Assuming the infections passed from the index to the contact, we observe the respective per-partnership transmission probabilities for Chlamydia and gonorrhoea in Table 2.

**Table 2:** Data from Lycke et al. [45] on case contact pairs for gonorrhoea and Chlamydia

	Male index		Female index	
Status of contact	No. of partnerships	% of partnerships	No. of partnerships	% of partnerships
Total	56	100.0%	47	100.0%
CT^1^+, GC^2^+	20	35.7%	12	25.5%
CT+, GC−	5	8.9%	1	2.1%
CT−, GC+	16	28.6%	24	51.1%
CT−, GC−	15	26.8%	10	21.3%
All CT+	25	44.6%	13	27.7%
All GC+	36	64.3%	36	76.6%

1 Chlamydia.

2 Gonorrhoea.

We assumed that both infections could have potentially passed from the index to the contact, but that neither infection influences transmissibility of the other, and that the infections were transmitted around the same time to the contact. Therefore, by using the per-sex-act transmission probabilities for gonorrhoea as in Table 1 (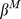 and 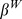), we can estimate the average number of sex acts that occurred in order to observe the above per-partnership probabilities of gonorrhoea transmission as 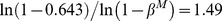 and 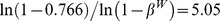 for the partnerships where the index case is male and female respectively. Then, to observe the respective per partnership transmission probabilities for Chlamydia with the corresponding number of sex acts, the respective per-sex act transmission probabilities for Chlamydia in those partnerships would thus be 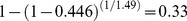 for males-to-females and 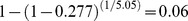 for females-to-males.

The above rests on multiple assumptions, but concurs with the opinion of various authors that Chlamydia is less transmissible than gonorrhoea [45], [47], [49], and was the best that could be done given the lack of direct estimates.

## Supporting Information

Figure S1 Figure S1A and S1B illustrate the classical model being calibrated to the output of the pair model for GC/CT (A) and HIV (B) respectively. The horizontal axes give simulation time in days while the vertical axes give *π*. For the same arbitrary partnership and gap lengths, the classical model is calibrated to give the same steady-state prevalence (*π^s^*) for GC/CT (A), and peak prevalence (*π^p^*) for HIV, as obtained from the pair model, with the direction of shift in prevalence from uncalibrated to calibrated as indicated by the arrow.
(TIFF)
