# Supplementary figures and images for: Comparability of Results from Pair and Classical Model Formulations for Different Sexually Transmitted Infections
Source: PLoS One. 2012 Jun 27;7(6):e39575. doi: 10.1371/journal.pone.0039575 (PMC3384672; doi:10.1371/journal.pone.0039575)

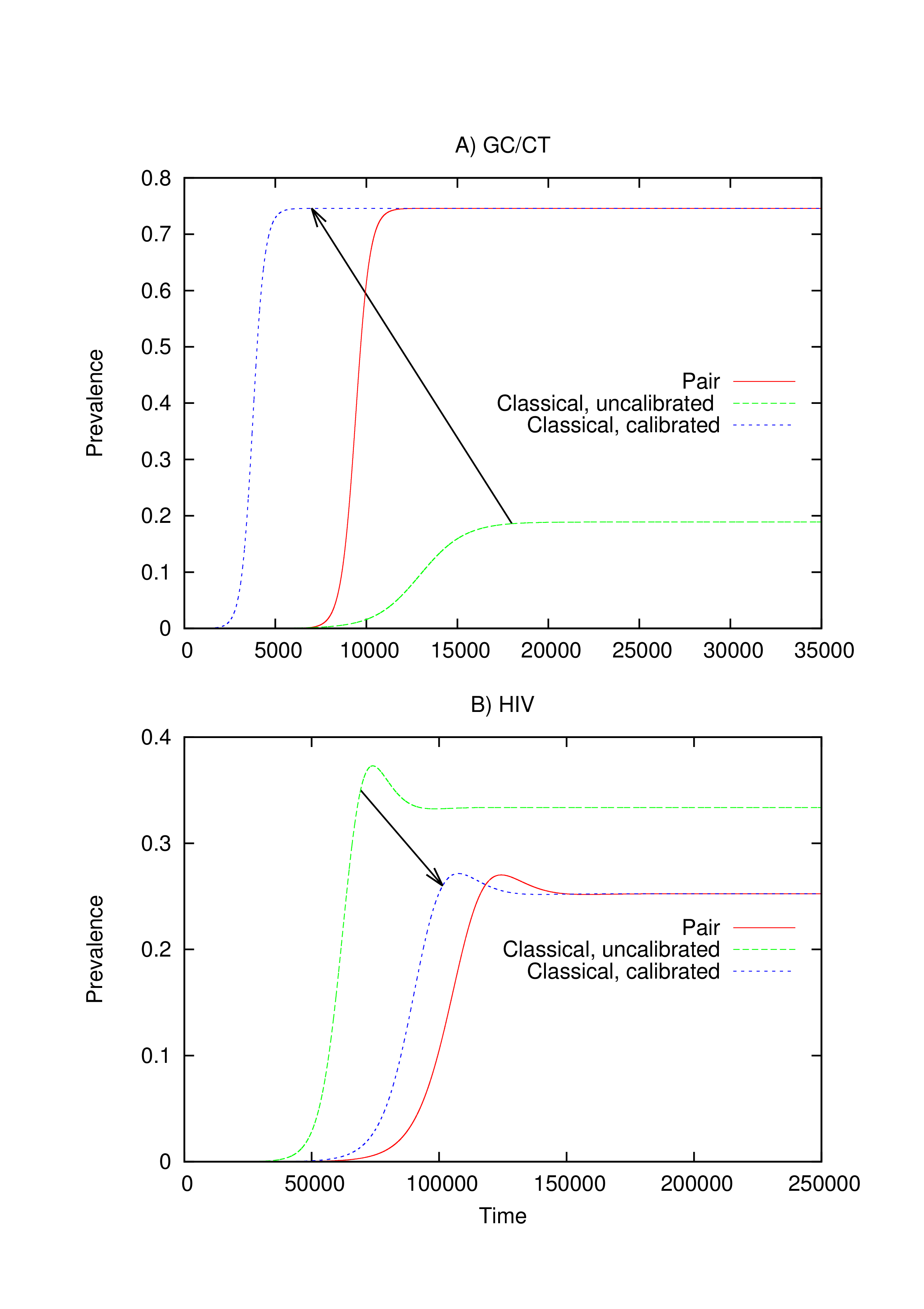

Supplement: Figure S1 — A and S1B illustrate the classical model being calibrated to the output of the pair model for GC/CT (A) and HIV (B) respectively. The horizontal axes give simulation time in days while the vertical axes give π. For the same arbitrary partnership and gap lengths, the classical model is calibrated to give the same steady-state prevalence (πs) for GC/CT (A), and peak prevalence (πp) for HIV, as obtained from the pair model, with the direction of shift in prevalence from uncalibrated to calibrated as indicated by the arrow. (TIFF) [file pone.0039575.s001.tif]
